# Material stiffness variation in mosquito antennae

**DOI:** 10.1098/rsif.2019.0049

**Published:** 2019-05-15

**Authors:** B. D. Saltin, Y. Matsumura, A. Reid, J. F. Windmill, S. N. Gorb, J. C. Jackson

**Affiliations:** 1 Centre for Ultrasonic Engineering, Department of Electronic and Electrical Engineering, University of Strathclyde, 204 George Street, Glasgow G1 1XW, UK; 2 Department of Functional Morphology and Biomechanics, Zoological Institute of the University of Kiel, Am Botanischen Garten 9, 24118 Kiel, Germany

**Keywords:** *Anopheles*, mating behaviour, *Toxorhynchites*, confocal laser scanning microscopy, finite-element modelling, antennal hearing

## Abstract

The antennae of mosquitoes are model systems for acoustic sensation, in that they obey general principles for sound detection, using both active feedback mechanisms and passive structural adaptations. However, the biomechanical aspect of the antennal structure is much less understood than the mechano-electrical transduction. Using confocal laser scanning microscopy, we measured the fluorescent properties of the antennae of two species of mosquito—*Toxorhynchites brevipalpis* and *Anopheles arabiensis*—and, noting that fluorescence is correlated with material stiffness, we found that the structure of the antenna is not a simple beam of homogeneous material, but is in fact a rather more complex structure with spatially distributed discrete changes in material properties. These present as bands or rings of different material in each subunit of the antenna, which repeat along its length. While these structures may simply be required for structural robustness of the antennae, we found that in FEM simulation, these banded structures can strongly affect the resonant frequencies of cantilever-beam systems, and therefore taken together our results suggest that modulating the material properties along the length of the antenna could constitute an additional mechanism for resonant tuning in these species.

## 1. Introduction

The exquisite sensitivity of animal sensory organs has been noted many times [1–6]. However, little attention has been paid to the mechanical properties that shape a sensor's response as much, if not more, as the neuronal filters [7]. The mosquito antenna is a well-known example of a highly sensitive particle-velocity receptor [2,3,8,9], and in many species, the key function of the antenna is to locate the flying conspecific mate [2,10–12]. Different models on how they achieve complex mechanical behaviour, for example, active amplification, have been proposed and are reviewed by Mhatre [13]. While knowledge of mechanical behaviour, for some sensory organs, has increased in the last decade [7], rather little is known about the material composition and properties underlying these complex behaviours in terms of the geometry-defining distribution of stresses and strains within the sensor [7].

**Figure 1.**
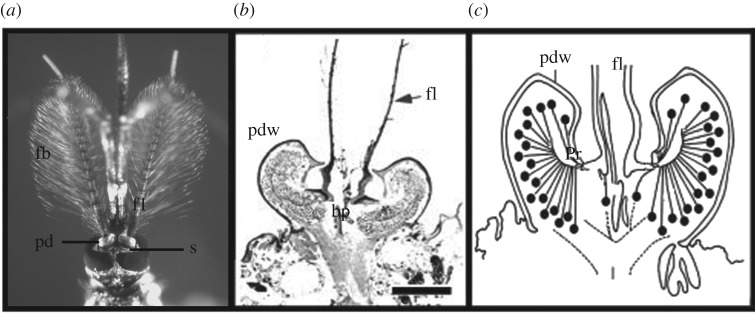
Schematic of antenna morphology. (*a*) Head of *T. brevipalpis*. (*b*) Cross section of the pedicel scale bar 0.1 mm. (*c*) Schematic of the inside of the pedicel (*b,c*) from Yack [15], simplified and modified. Image abbreviations: bp, basal plate; fb, fibrillae; fl, flagellum; pd, pedicel; pdw, pedicel wall; pr, prongs; s, scape.

The mosquito antenna comprises three parts—scape, pedicel and flagellum [1] (figure 1). Control of the antennal direction is done in part by the scape, but the scape is not relevant to the present study. The pedicel houses some 16 000 sensory neurons [14], the majority of which are used for acoustic detection. These neurons connect to radially distributed prongs, attaching the neurons to the base of the flagellum. The flagellum itself is the physical sensor—it consists of 13 sequential flagellomeres that project distally. These act as viscosity sensors, undergoing oscillatory displacement in the presence of acoustic fluid flow. The flagellum is covered in hair-like structures known as fibrillae (or setae) in which case the antenna is considered *plumose—*these fibrillae serve to increase the viscosity of the sensor improving its performance [2]. It has been shown that for biologically relevant sounds, the flagellum moves like a paddle [2]. Taken together, the whole system is resonantly tuned to respond maximally at the wingbeat frequencies of flying conspecifics and is the key sensor in the animal's phonotactic mating behaviour [11].

Most research into mosquito audition focuses on the biophysical basis for mechano-electrical transduction that endows these sensors with high sensitivity and often assumes that the flagellum is simply a stiff rod in which the material properties are of negligible importance. However, we consider material properties to be an overlooked and potentially important factor in the biophysics of acoustic perception. As the resonance of the whole system will undoubtedly be determined by some contribution of the flagellum and the effective stiffness of the pedicel attachment, it is possible that the way in which the flagellum is built may be significant for resonance tuning, and thus mating behaviour. Therefore, with no *a priori* expectation on the spatial distribution of material properties, we investigated the stiffness distribution in mosquito antennae with confocal laser scanning microscopy (CLSM).

The use of CLSM for obtaining information about general types of cuticle present is well established (e.g. [16–24]). It enables us to visualize the insect exoskeleton using autofluorescences and to estimate material properties of structures. Michels & Gorb formulated that it is capable of estimating material properties, since well-sclerotized, flexible and resilin-dominated regions are visualized differently [16]. It has also been previously cross-validated using three-point bending tests, AFM nanoindention and compression tests [17,22,23]. Merits of CLSM are that the technique allows one to take sharp images of narrow sample planes by restriction of light entering the camera. This is achieved by the appropriate choice of pinhole size depending on the wavelength of the light, creating optical sections of the object. This image stack in turn then can be combined to create maximum intensity projections—in one image—showing the structure without loss of depth resolution (electronic supplementary material, table S1). One can therefore image the whole intact surface structure in great detail. In addition, the varying excitability of different unknown cuticle compounds with different laser wavelengths provides an estimation of material properties of the cuticle.

In many mosquito species, acoustic communication is essential for mating [10–12,15] and many are vectors for animal and human diseases, such as malaria, yellow fever or the zika virus [12,25]—indicating how important it is to understand their biology.

Antennae of two mosquito species of different size and ecology—*Toxorhynchites brevipalpis* and *Anopheles arabiensis*—were examined here. Many *Anopheles* species are swarming mosquitoes in which acoustics is crucial during mating [10,11,26,27]. However, *T. brevipalpis* is a solitary non-swarming species, in which their behaviour is scarcely documented [28,29].

There is significant interest in the *Toxorhynchites* genus for mosquito population control as its larvae predate on other mosquito larvae, many of which are species that have global importance [28,29]*.* As this mosquito is large, robust and non-biting, it has been previously used as an amenable model system in earlier studies of insect auditory systems [8,9,26,30]. According to Gibson & Russell [30], the wingbeat frequency of males and females synchronize during aerial mating [12,26]—and this is termed distinct flight/wingbeat frequency-matching during the mating display. As antennal ears are part of detecting the mating partner, and beam-like structures are most sensitive around their resonance, it is beneficial to have the resonance of the sensory organ close to the acoustic stimulus (the wingbeat frequency) [31]. Similar acoustic behaviour has been observed in *Anopheles*, which are responsible for the spread of diseases in cattle and humans, and therefore play a substantial social and economic role [27].

Neither of the two species mentioned above have, to our knowledge, been studied for the potentially varying stiffness along the length of the flagellum. As we will show, CLSM proves to be very useful in demonstrating changes in material stiffness along the flagellum. From the results of the CLSM work detailed later, we used finite-element modelling (FEM) to determine the effect of the measured stiffness profiles along the antenna on a compliantly clamped beam.

## 2. Material and methods

### 2.1. Specimen preparation

Animals were deeply anaesthetized with CO_2_, and dissected in PBS (Carl Roth GmbH & Co KG, Karlsruhe, Germany). The antennae were treated with the surfactant Triton X-100 (Sigma-Aldrich Chemie GmbH, Steinheim, Germany), to ensure wetting of the entire surface, a necessary step as the fibrillae of plumose antennae easily trap air bubbles. Triton X-100 was washed off in triple steps with PBS. Antennae or their parts were transferred to glycerine (Carl Roth GmbH & Co. KG, Karlsruhe, Germany), which is a suitable medium as it has a similar refractive index to glass [16].

In the present study, only males are included. There are three main reasons for this: (1) due to strong structural sexual dimorphisms, morphological sex comparisons are difficult. (2) While acoustics is important to both sexes (e.g. [30,32]), the male antenna is the most studied with respect to their acoustic response, lending itself to easier comparison. (3) CLSM as relative method benefits more from comparing two separate, but structurally similar objects. Four individuals of each species were used for the present study, not all of which were CLSM-imaged, but it was confirmed the structures imaged are typical, either under the CLSM Zeiss LSM 700 (Carl Zeiss Microscopy GmbH, Jena, Germany) or a fluorescence microscope (Zeiss Axioplan).

### 2.2. CLSM operation

Following an established standard method [16], which is applied for many insect exoskeleton studies (e.g. [33–35]), antennae were visualized using autofluorescences using the same excitation wavelengths and emission filters applied in the referenced studies. Michels & Gorb [16] demonstrated that the resilin autofluorescence is excitable at the wavelength of around 405 nm. Moreover with a combination of different excitation wavelengths, the method can estimate differences of material properties within a properly adjusted image or images taken with same settings, because in general well-sclerotized, flexible and resilin-dominated regions are visualized differently [16]. The stack construction was performed with ZEN 2009 (6.0 SP2) (Carl Zeiss MicroImaging GmbH), which automatically corrects for wavelength-dependent slice thickness and resulting in varying overlap between stacks taken with different wavelengths.

After adjustment of excitation level according to existing standards [16], the specimens were exposed sequentially to four different excitatory wavelengths (405, 488, 555 and 639 nm), and emitted lights were filtered using a band-pass emission filter of 420–480 nm and long-pass emissions filters transmitting light with wavelengths ≥490, ≥560 and ≥640 nm, respectively.

Each sample was imaged only once to avoid the photobleaching which could alter the resulting visualization of the material, as different compounds are not similarly susceptible to this effect. This is important for our case, since relatively high laser power (up to max. 30%) was used in order to allow weakly fluorescent structures to be imaged. High laser power can lead to pronounced bleaching if the sample were to be imaged multiple times with the same laser [16].

### 2.3. Image colour coding

Confocal laser scanning micrographs were colour-coded according to Michels & Gorb [16]. Blue, green and two red image colours were assigned for each micrograph corresponding to excitation wavelengths 405, 488, 555 and 639 nm, and filters 420–480, ≥490, ≥560 and ≥640 nm, respectively. The two ‘red’ channels, which provide similar results, were each set to 50% saturation and combined into one red channel to compensate for the double acquisition, a process required as otherwise the red autofluorescence would unduly dominate the image making assessment difficult. According to [16], material properties were interpreted based on resultant micrographs as follows—in superimposed images of insect exoskeletal parts: (1) well-sclerotized structures are usually red, (2) tough-flexible cuticular structures are typically yellow-green, (3) relatively flexible parts containing a relatively high proportion of resilin are light blue and (4) resilin-dominated regions appear deep-blue. Note that the image colours represent autofluorescence and code the intensity of light in specific channels.

This interpretation was confirmed by observation of corresponding samples under a stereomicroscope. Since assessment of material based on CLSM are relative, not absolute, the comparison is only valid if images are taken with the same settings (electronic supplementary material, figure S1 and table S1), or if two structures are imaged in one single scan (figure 3*b,c*).

Therefore, when necessary we scanned multiple samples simultaneously. Images are reproduced here with increased brightness and contrast, to improve clarity (unaltered images are available in the electronic supplementary material). The exact imaging settings can be seen for each image in electronic supplementary material, table S1.

### 2.4. Finite-element modelling

FEM with COMSOL 5.3a (Comsol Inc., Stockholm, Sweden) was conducted in the frequency domain to investigate the vibrational characteristics of the antennae using the Solid Mechanics module. Simulations were performed on both desktop computers and the ARCHIE-WeSt supercomputer.

The objective of these simulations was to ascertain the relevance of the observed banded structure of the flagellomeres on the overall frequency response of an idealized compliantly clamped beam. We have reduced the mosquito antenna to a simple system, and thus the simulations are solely to observe the frequency-response changes due to the presence of hard and soft rings in the flagellomeres.

Control simulations were done on uniform cylinders whose expected resonant frequencies are well established from Euler–Bernouilli beam theory. Once established that control simulations yield appropriate results, a subdivided ringed structure seen in the CLSM images was simulated (figure 3).

The fibrillae were not included in the model—they are apparently stiffly coupled to the beam, so that for biologically relevant frequencies, the fibrillae and flagellum move as one [2]. From a mechanical perspective, these fibrillae add damping, but little mass, and thus broaden the response of the whole antenna but do not shift the resonance frequency appreciably. Additionally, the densities of the materials modelled are identical, since remarkable density differences are not known despite the range of stiffnesses in chitinous structures [36].

The geometry of the model antenna was informed by the images that indicated that the cuticle of the flagellum is a thin sheet compared to the absolute volume, hence the flagellum is represented by a 10%v wall hollow cylinder filled with tissue (90%v, Young's modulus 1 kPa), which we will describe as a beam, to avoid confusing it with the biological structure. The tissue was assumed to have a fraction of the literature value for soft tissue as those values are given for pulling on tissue assemblies in the direction of maximum resistance.

**Figure 2.**
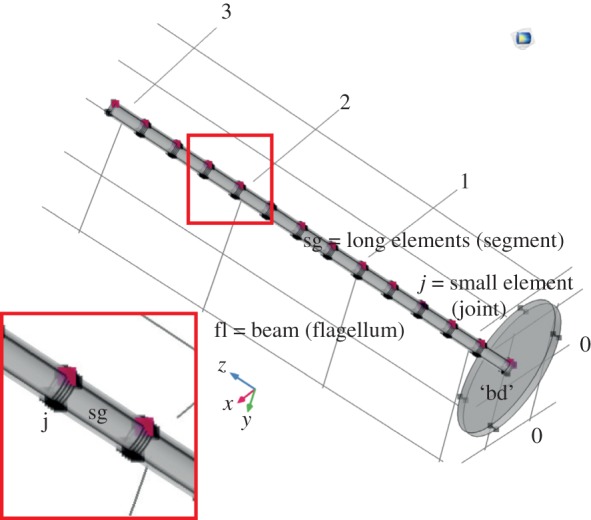
Three-dimensional representation of our antenna model. This figure correlates modelling terminology with morphological terms. The antenna comprises 13 segments with 12 joints. In a model for *T. brevipalpis*, the antennal structure is a sequence of relatively hard long elements, interspersed with a series of three small elements of one soft material sandwiched between two hard small elements (figure 3*a,b*). In contrast in *Anopheles*, the antennal structure is a sequence of soft elements, separated by thin discs of hard material (figures 3*c,d* and 4). Points used for simulations in figure 6 are shown in red. To simulate an impinging sound field, a load was applied perpendicular to the beam axis in the +X direction on elements 2–13. Image abbreviations, with morphological terms in brackets if applicable: ‘bd’ basal disc (approximating the pedicellar articulation); fl, beam (flagellum); j, three small elements (joint); sg, long element (segment).

This beam, illustrated in figure 2, was subdivided into 13 long elements of constant size separated by 12 triplets of small elements, to represent the segment joint areas of the antenna. Size of the individual elements was 1/13 of the antenna length (3.3 mm for *T. brevipalpis* and 1.7 mm for *An. arabiensis*) and the diameter was 120 µm for *T. brevipalpis* and 15 µm for *An. arabiensis*. Of importance for the present study is ensuring the spatial distribution of elements of different stiffnesses was matched to the results of the CLSM imaging.

**Figure 3.**
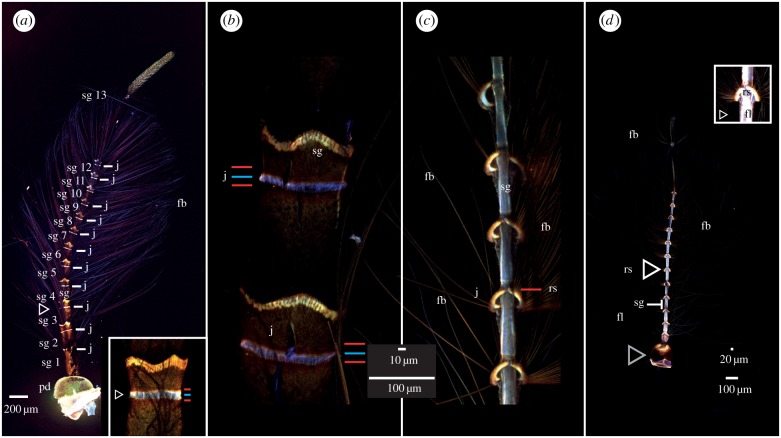
CLSM-based maximum intensity projections of the male antennae of *T. brevipalpis* (*a,b*) and *An. arabiensis* (*c,d*). Higher resolution images available in the electronic supplementary material. (*a*) Antenna, with increased brightness, showing the 12 more-or-less regular subunits and a varying 13th one. Inset: Zoomed image of bands of a different individual. (*b,c*) Comparison image of (*b*) *T. brevipalpis.* (2nd segment) and (*c*) *An. arabiensis* (4th to 8th segment), with increased brightness. The antenna of *T. brevipalpis* is larger and thicker. It is made up of relatively stiff cuticle with small, relatively flexible (blue) and hard (red/orange) rings, while *An. arabiensis* antenna is made up of relatively soft (light blue) cuticle interspaced with hard rings. In both species, the area where the fibrillae emerge is hard (orange). In (*a,b*) (*T. brevipalpis*), the image shows two red-orange discs sandwiching a blue disc at the base of the segments (white arrow). (*d*) Overview of *An. arabiensis* with increased brightness. The overall anatomy is dominated by relatively soft and flexible areas, interspaced with comparatively hard bands where fibrillae insert. Image abbreviations: fb, fibrillae; fl, flagellum; j, joint; pd, pedicel (grey arrowhead in (*d*)); rs, ring structure; sg, segment.

**Figure 4.**
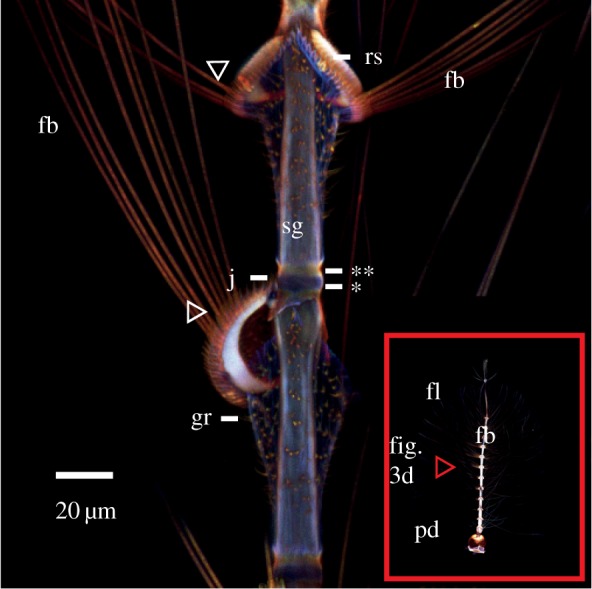
Maximum intensity projection of *An. arabiensis*, with increased brightness, showing detail of two flagellomeres and the insertion position of fibrillae. Males of this species can erect the fibrillae depending on diurnal cycle and activity, due to the presumably soft, sac-like structure that is outlined by the granulae (**gr**) below the hard ring structure (**rs**). The combination of these two structures can potentially provide the mechanical basis to inflate by hydraulic pressure and erect the fibrillae. Fibrillae sockets on the ring structure (white arrowheads) are where the fibrillae insert. Image abbreviations: fb, fibrillae; fl, flagellum; gr, granulae; j, joint; pd, pedicel; rs, ring structure; sg, segment. *Deep-blue fluorescent small band, **yellow-orange fluorescent band.

The articulation in the pedicel, which unquestionably contributes to the mechanical behaviour of the antenna, is represented by the round disc (figure 2) for simplicity and is modelled with an effective stiffness that takes into account the diameter of the prongs and an approximation of their stiffness. This compound quality of the pedicel articulation is later on referred to as ‘basal stiffness’. It is important to note that our ‘basal disc’ is not matched in size or any other way to the basal plate, but its allometric relation to the beam length and diameter ensures it stays constant in relative size between species.

As estimations of Young's modulus of insect cuticle vary greatly between different reports (see [36,37] as examples), only the ratio of Young's moduli between the materials was used for simulations, while keeping values in the natural range. The large and small segments were allocated stiffnesses within the range of the material property estimation according to CLSM images and literature values [36].

Taken together, the model beam is as follows: for *T. brevipalpis* the large elements are of medium stiffness (0.5 GPa) and the triplet of small elements is a stack of hard (5 GPa), soft (1 MPa) and hard element (5 GPa), followed by the next ‘large element’. For *An. arabiensis* the large elements are soft (1 MPa) and all three of the triplet of small elements are hard (5 GPa). Vibrational characteristics of the modelled antennae were simulated in 10 Hz steps (figure 6) over the frequency range of 10–2720 Hz, which includes the typical hearing range of these insects. Only the range 20–2000 Hz is shown.

## 3. Results

Results are presented separately for each species and highlight differences and similarities in antennal structure as well as its putative material properties. According to the colour scheme of [16], areas shown in blue are likely to be resilin-dominated structures, relatively soft structures will appear light blue, tough structures in yellow-green and sclerotized structures in red.

### 3.1. *Toxorhynchites brevipalpis* male

The flagellum consists of 13 segments, each of which, except the first that articulates in the pedicel and therefore cannot be observed directly, has a blue ring followed by a yellow-reddish arrowhead or coronal structure that appears to be more sclerotized. At the tip of this structure, fibrillae emerge in a pattern shown in figure 3*a,b*. Distal to the coronal structure, a comparatively weakly fluorescent material is located. The thin final ring-like structure, observable in detail in red, is interpreted as being part of the lower segment (figure 3*b*), and the next segment therefore begins with a blue ring structure. The segments continuously decrease in length and diameter from proximal to distal locations.

By contrast, the fibrillae have a relatively uniform length and autofluorescence along the flagellum. The long fibrillae are absent on the 13th segment, giving the antenna a somewhat rounded appearance (figure 3*a*). The 13th segment is different from the others; it continues for five to six times the length of the 12th, is noticeably thinner, and shows little tapering.

**Figure 5.**
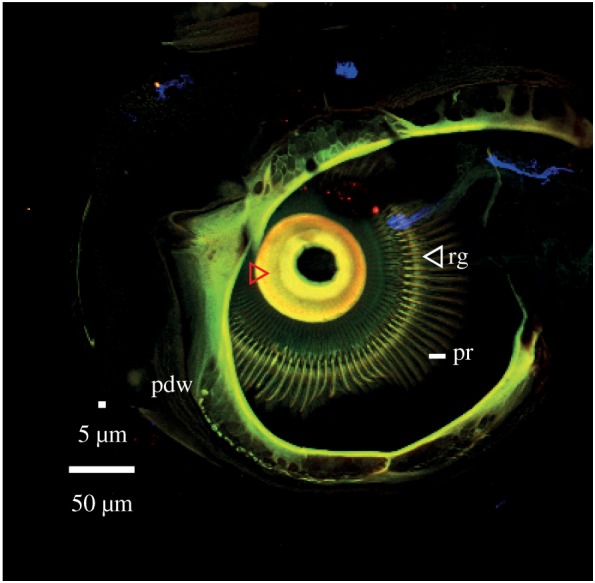
CLSM images of a *T. brevipalpis* male that shows a maximum intensity projection of an opened pedicel. This attachment is comparatively hard (red arrowhead) and the prongs run over a ridge (white arrowhead), which appears as hard as the prongs themselves. Image abbreviations: pdw, pedicel outer wall; pr, prongs; rg, ridge (white arrowhead).

The 13th segment only sprouts a limited amount of much-shorter fibrillae (figure 3*a*). Externally, the pedicel autofluorescence is rather uniformly green, except the uppermost ridge, where the flagellum emerges (figure 3*a*). Where the pedicel and flagellum join, the material appears orange, due to a higher contribution of red autofluorescence compared to the pedicel's outer wall (figure 5 red arrowhead and electronic supplementary material, figure S1a).

The inside of the pedicel is dominated by centrally attached prongs. Note that the prongs are uniform in their green autofluorescence (and thickness) with their neighbours, as far as can be assessed (figure 5 and electronic supplementary material, figure S1), indicating that prongs are of uniform stiffness.

An optical section (electronic supplementary material, figure S1a) of the pedicel indicates that the prongs either attach to, or pass over, a cuticular ridge. The thickness of the whole articulation point is less than approximately 2 µm (electronic supplementary material, figure S1).

However, it should be noted that across individuals and depending on experimental settings, the colour of autofluorescence varies—the difference in settings between experiments makes comparison across individuals difficult.

### 3.2. *Anopheles arabiensis* male

Segments 2–12 are very similar, albeit gradually tapering to about half the initial diameter by segment 12. Each segment consists of a blue part (figures 3 and 4), followed by a broadened ring-like sclerotized structure in orange-brown-red circumventing the flagellum from which the fibrillae crest of each segment emerges (figure 3*d* white arrowhead figure 4). The first segment is of similar structure, but along the flagellum axis, the segment first tapers after insertion, then becomes medially swollen (figure 3*d*). The 13th segment is filamentous (figure 3*c*), showing overall low autofluorescence.

Details of the more strongly orange-red fluorescent ring structure include the socket that each individual fibrilla inserts into (figure 4 white arrowheads). At the site where the ring joins the flagellum, a deep-blue fluorescent small band of similar width as the ring is present (figure 4*), followed by a more yellow-orange fluorescent band (figure 4**) before the segment continues to show a light blue material fluorescence. Note that the area proximal to the ring structure and surrounding the flagellum is lacking autofluorescence, with the exception of scattered orange fluorescent granulae which outline the cuticle sac area (figure 4, gr below rs). The rather sclerotized articulation of the flagellum in the pedicel is approximately hemispherical and at the upper end of the distalmost part of the pedicel (figure 3*d* grey arrowhead). With CLSM, further internal structure, except the crest of uniformly fluorescent prongs, cannot be visualized (electronic supplementary material, figure S1b).

### 3.3. *Toxorhynchites brevipalpis* and *Anopheles arabiensis* male comparison

Comparison of the second segment in *T. brevipalpis* (figure 3*b*) to the sixth to 10th segments of the antenna in *An. arabiensis* (figure 3*c*) shows a noticeable difference in size. These segments in *T. brevipalpis* are 120–150 µm wide and 200 µm long. The antenna of *An. arabiensis* is much smaller with each segment about 100 µm long, and only 10–20 µm wide. The long fibrillae inserting on the orange structure circumventing the flagellum lack an autofluorescence gradient and are barely visible in both species as autofluorescence is overall low in the fibrillae.

Whereas large parts of *T. brevipalpis* show nearly no signal in contrast autofluorescence in *An. arabiensis* is in general stronger, every part of the antenna is either likely flexible (blue) or putatively sclerotized (orange) with no areas of intermediate properties. As a further difference, it can be stated that the most red part in *T. brevipalpis* is a coronal structure, while it appears more annular in *An. arabiensis* (figure 4). Both antennae are almost mirror images in the respect that in *T. brevipalpis* a rather small band (*ca* 10 µm) of a slightly deeper shade of blue is in between each segment, while the whole 100 µm long segment in *An. arabiensis* shows light blue autofluorescence. An antennal cross section shows that a rather thin surface layer constitutes the highest proportion of the blue autofluorescence (electronic supplementary material, figure S2) in both species.

Furthermore, the optical sections (electronic supplementary material, figure S1) show that the articulation structure remains uniformly thin (*ca* 2–4 µm) and comparable in size between the two species*,* despite the fact that the pedicel of *An. arabiensis* is roughly half as large as the pedicel of *T. brevipalpis*.

### 3.4. FEM of the male antenna

**Figure 6.**
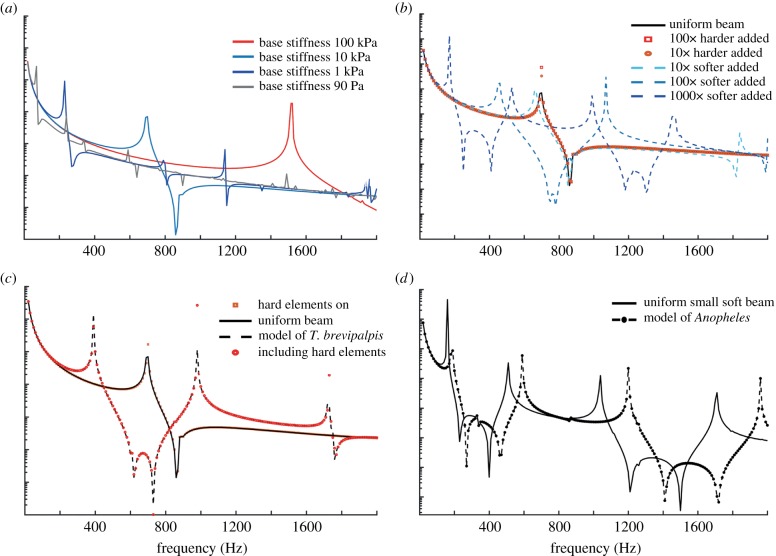
Simulation results. For a modelled beam, (*a*) shows the change of frequency response over four orders of magnitude of base stiffness, and (*b*) shows the effect of adding hard or soft elements to a uniform beam with base stiffness of 10 kPa as in figure 2. Overlaid are dashed curves indicating simulations where both hard and soft elements are added—in this case 10× harder elements in combination with 10×, 100× and 1000× softer elements—colour-coded as for soft elements. (*c*) Comparison of a *Toxorhynchites*-type model with either uniform stiffness, or hard and/or soft elements added. Only the addition of soft elements appreciably affects the frequency response. Note that the resonance is consistent with wingbeat frequencies in this species. (*d*) Comparison of the *Anopheles*-type model with either uniform stiffness or the addition of hard elements. Hard elements increase the resonant frequency.

Frequency-domain studies were performed in COMSOL to observe the effect of the various segmental structures seen in CLSM experiments, as well as other standard parameters of an antennal system such as ‘base stiffness’ and geometry. Figure 6*a* demonstrates the effect of changing ‘base stiffness’ on the frequency response of a simulated beam. It is clear that increasing basal stiffness leads to an increase in the frequency of the resonant peaks. As expected, further increases in basal stiffness yield diminishing changes in the frequency response as predicted by equation (4.1). In the investigated stiffness range for the base of 90 Pa (70 Hz)–100 kPa (1520 Hz), a diminishing gain per decade stiffness increase is observed.

In Figure 6*b*, elements of different elasticity were added to a beam, distributed in agreement with the CLSM results. It is clear that the stiffness modification of segmented structures in the antennal model influences resonant frequencies.

For the beam simulated in Figure 6*b*, softer segments are shown to reduce the peak frequencies by 35, 240 and 525 Hz as we decrease the stiffness by powers of 10. By contrast, the addition of harder elements leads to an increase in the peak frequencies, although to a lesser extent. Without hard elements the resonant frequency is between 690 and 700 Hz and with hard elements the resonant frequency shifts up to 700 Hz.

While figure 6*a*,*b* is already dimensionally matched to *T. brevipalpis,* figure 6*c*,*d* shows simulations of frequency responses in male *T. brevipalpis* and *An. arabiensis,* respectively*.* The graph in figure 6*c* shows four lines associated with four cases: (1) uniform beam, (2) uniform beam with added stiff elements, (3) uniform beam with added soft elements and (4) uniform with both stiff and soft elements. The latter case is the most morphologically accurate. Clearly, the addition of soft elements is the only case which allows for significant changes in resonant frequencies, from approximately 695 Hz down to 390 Hz. By contrast, the addition of hard elements is insufficient to affect the natural vibration characteristics substantially, resulting in under a 10 Hz shift from approximately 695 Hz. In figure 6*d*, the two lines represent: (1) a uniform small and soft beam of the same size as the *An. arabiensis* antenna and (2) the same beam but with added hard elements. The latter case is most faithful to the antenna of *An. arabiensis.* The frequency shift appears to be less than the 305 Hz shift shown in figure 6*c* for soft elements in a harder beam. The addition of hard elements increases the resonant frequency of the overall softer beam from 160–190 Hz. While figure 6*a* demonstrates the model representing the situation in a compliantly clamped beam, figure 6*b–d* demonstrates that distribution of varying material properties can have a strong effect on the overall resonant frequency. By contrast, in a simple compliantly clamped beam, the resonant frequency is mostly determined by the beam dimensions and the stiffness of the clamp [38].

Figure 6*c,d* shows simulations based on stiffness ranges obtained from CLSM autofluorescences in the two species, as well as appropriate geometry. The example of *T. brevipalpis* demonstrates how it is possible to reduce the resonant frequency of a beam to match the observed frequencies seen in the insects studied (i.e. in *T. brevipalpis* and *An. arabiensis* resonant frequencies are 420 ± 5 Hz [39] and 380 ± 46 Hz (new experimental data not shown) for the males, respectively). It is worth noting that within biologically relevant parameter ranges for antenna stiffness *without* adding disc elements to the beam, it is difficult to obtain resonant frequencies in simulation that match those seen in nature. Taken as a whole, the addition of elements of different stiffness in comparison to the main beam can change beam resonant frequency to a similar order of magnitude as changing the basal stiffness (figure 6*a*).

## 4. Discussion

The goal of this work was to investigate the material properties of the mosquito antenna. We performed a CLSM study of mosquito antennae and found that autofluorescence is not homogeneous along the antenna, but instead these antennae comprise repeating bands of harder and softer elements. In general, the presence of harder and softer elements in the antenna is similar between the two mosquito species—however, their distribution is inverted: *An. arabiensis* has large rather flexible bands interspaced with harder ring elements, while *T. brevipalpis* is medium-hard overall and has short flexible ring elements wedged between two hard rings. Given the nature of the results, we also simulated these geometrical configurations to determine whether these material changes and spatial distribution has any effect on the overall resonant frequency of the antenna, a property of significant importance to the animal, as sensitivity of these ears is best around the resonant frequency and acoustic perception is essential for mating [10,11,26,27,30,31]. It is possible that the different sizes of the animals studied may influence the material properties of the antennae, as the larger antennae perhaps have different mechanical constraints to ensure robustness and structural stability. It is not clear in general whether the observed geometrical differences are driven by behavioural or other constraints unique to each species. These questions could be answered with studies on similarly sized and more behaviourly similar species, and are not addressed here. Some interpretation on how the observed stiffness distribution along the modelled beam could play a role in the resonant characteristics, and the implications of this, might be inferred from FEM simulations discussed below.

### 4.1. Confocal laser scanning microscopy

In *T. brevipalpis* males*,* the larger of the species investigated, the overall structure is relatively tough and there exist small flexible to well-sclerotized ring structures. In *An. arabiensis* males*,* the combination of a sclerotized ring structure, on which the fibrillae are present in each segment, and the area of the membrane sac proximal to it is believed to play a role in the behaviour of male *Anopheles* antenna, in which they collapse and extend their fibrillae at different times of the day using hydraulic pressure [40]. Our images are taken after suspension in fluid and show only antennae with extended fibrillae. This position in nature is only assumed during the active swarming phase [40] and therefore is directly related to the detection of conspecifics. Pedicels both internally and externally look similar in structure, but in *An. arabiensis* the intensity of autofluorescence is higher. In male *T. brevipalpis,* a hard area (figure 5 red arrowhead) is visible, where the flagellum leaves the pedicel. The prongs on the inside appear similar in the two species regarding fluorescence and dimensions compared with prongs of the same animal in the same image. Comparison between images demonstrates that different CLSM settings are necessary for proper visualization of material differences this does not allow judgement of material properties between species imaged individually. However, we can say with some confidence that the prongs are neither particularly flexible nor stiff and are all consistent in their autofluorescence and dimensions within the animal.

This is in agreement with Avitabile *et al*. [41], in that the prongs act more or less as rigid-body extensions of the flagellum. Possible variations inside the pedicel would likely be due to the scolopidia, which have recently been shown, by direct measurement using atomic force microscopy, to be motile [42], having long been suspected as the source of stiffness gating. Further studies have shown the importance of the scolopidia for both power gain of the antenna, and the intra- and interspecifc variations seen in antennal mechanics [32].

However, the present study demonstrates that the flagellum itself cannot be approximated as a rigid beam of uniform stiffness, but that it consists of repeating units of stiff and soft elements. A limitation of the present study is the lack of direct correlation of CLSM-based autofluorescence analysis with mechanical measurements, which is to be tackled in follow-up investigations.

### 4.2. Significance of material property differences in antennae

Since Johnston (1855) [1], the nerve and cuticle structure inside the pedicel has been investigated regarding its auditory and general function. In this study, we visualized the antenna of *An. arabiensis*, a species where males form swarms and females fly into the swarm and are acoustically located by the males. *Toxorhynchites brevipalpis* is a solitary mosquito, where acoustics also plays a role in mating [30]. Within individuals, consistency of the uniform prongs inside the pedicel has been found across both species, and remarkable differences in material distributions were found in the flagellum.

A sensory organ exposed to the environment such as an insect antenna (that can move autogenously without stimulus) is potentially under continuous mechanical stress. This may explain the presence of resilin, a protein known to be used to protect from ‘wear and tear’ in insects [43,44].

The FEM results indicate a potential for resonant tuning by alteration of material distributions along the flagellum. This is perhaps not that surprising, as the influence of differently stiff elements partially can be expected as the resonant frequency (*ω*/*2π*) of a beam [38] is dependent on Young's modulus through

4.1 $$\omega =k^{2}\sqrt{\frac{EI}{\rho A}}\;,$$

where *k* is the wavenumber, *I* is the area moment of inertia, *ρ* is the density, *A* is the cross section of the beam, and *E* is Young's modulus. In a composite beam, Young's modulus would likely be an effective Young's modulus of the whole beam, which will be different when elasticity is not uniform. Furthermore, the non-homogeneous material distribution as suggested by CLSM results along the antenna could affect the area moment of inertia. Given the densities are suspected to be similar, the likelihood of this being important is low. Regardless, by changing the distribution of stiffnesses, a further mechanism to control the resonant frequency of the antenna is possible. There are potential benefits to this mechanism in conserving structural integrity in comparison to changing the material.

FEM models of material property distributions in the antennae of the studied insects have been compared to a uniform beam structure. This shows different mechanical behaviours, suggesting that a more rigid antenna, presumably like that of *T. brevipalpis* (figure 6*a*), and a soft antenna, like that of *An. arabiensis* (figure 6*b*), can both be tuned significantly by basal stiffness and distribution of stiffness along the beam. However, the effect of hard elements in soft beams seems less than that of soft elements in hard beams. How this different tuning affects behaviour requires further research.

Interestingly, in order to make our model show resonant frequencies that are found in these insects, the addition of the triplet rings was essential. We found it difficult to reproduce the resonant frequencies found in *T. brevipalpis* and *An. arabiensis* (420 ± 5 Hz [39] and 380 ± 46 Hz (data not shown) for the males, respectively) using only the known geometry and typical biological values for material properties—it was somewhat surprising that only the addition of the triplet rings allowed one to bring down the resonant frequencies in *T. brevipalpis* to observed values. The deviation in *An. arabiensis* is due to the other contributing factor—basal stiffness.

Our FEM model shows a very weak first bending mode at the main resonant peak and a pendulum mode at lower frequencies. In particular, in the case of a small angular displacement, the weak bending mode can easily be perceived as a pendulum mode, as while tip displacement is largest, displacement overall is fairly uniform in both of these mode shapes. This is in line with earlier experiments in different species reporting a pendulum mode [2,7].

Generally speaking, there are many factors that may contribute to the antennal mechanical behaviour. An exhaustive list would include the stiffness of the base articulation, the cell attachments to the scolopidia, prongs and scolopidia, geometry, and viscous effects of the fibrillae, among other things. For example, it has been shown in stick insects that tapering of their non-plumose antennae has the largest tuning effect, at least in the static case [44]. The current study suggests that the configuration of spatial distribution of flexibility along the antenna does influence the antenna's mechanical behaviour within the frequency range of the animal's hearing. More in-depth experimental and theoretical investigations of antenna bending are required, which will add to our understanding of mechanical properties of insect hearing systems.

## 5. Conclusion and outlook

Generally, the highly variable and interesting material properties of insect cuticle have not gone unnoticed (e.g. [21,45,46]). However, in the field of insect auditory systems, the fine detail of material properties and distribution is overlooked. The primary goal of this work was to observe and describe morphological and material differences and similarities in antennal hearing organs of the investigated species. While it is possible to consider the antenna as a simple beam, we have shown that the actual material properties of these antenna are more complex. We also show that these material complexities have the potential to modify the frequency responses of the acoustic sensors, providing a different mechanism to the animal to evolve and direct—mating-critical—frequency selectivity. The addition of soft elements to a hard beam shifts the resonant frequencies to lower values, while adding stiff elements to a soft beam does the opposite and shifts the frequency of resonant upwards. The study of varying material distribution of insect hearing organs with CLSM has a high potential for improving our understanding of the evolution and development of acoustic sensors in nature, especially if combined with FEM and possibly mechanical tests of materials and laser vibrometry, to characterize native system behaviour. The very high variability of mechanical and therefore acoustic properties in these insects studied suggests a potential for many interesting future findings and biomimetic engineering exploitation.

## Supplementary Material

Figure S1

## Supplementary Material

Figure S1a

## Supplementary Material

Figure S1b

## Supplementary Material

Figure S2

## Supplementary Material

Figure S3a

## Supplementary Material

Figure S3a - high resolution

## Supplementary Material

Figure S3b&c

## Supplementary Material

Figure S3d

## Supplementary Material

Figure S4

## Supplementary Material

Figure S5

## Supplementary Material

Table S1

## Data accessibility

Additional images, all original images and image values (electronic supplementary material, table S1) are available on (https://figshare.com) via Interface. Additionally, data is freely available from the University of Strathclyde KnowledgeBase at https://doi.org/10.15129/8ec4ac84-3040-4c4d-854e-07e50786a68a.
