# Supplementary figures and images for: Material stiffness variation in mosquito antennae
Source: J R Soc Interface. 2019 May 15;16(154):20190049. doi: 10.1098/rsif.2019.0049 (PMC6544878; doi:10.1098/rsif.2019.0049)

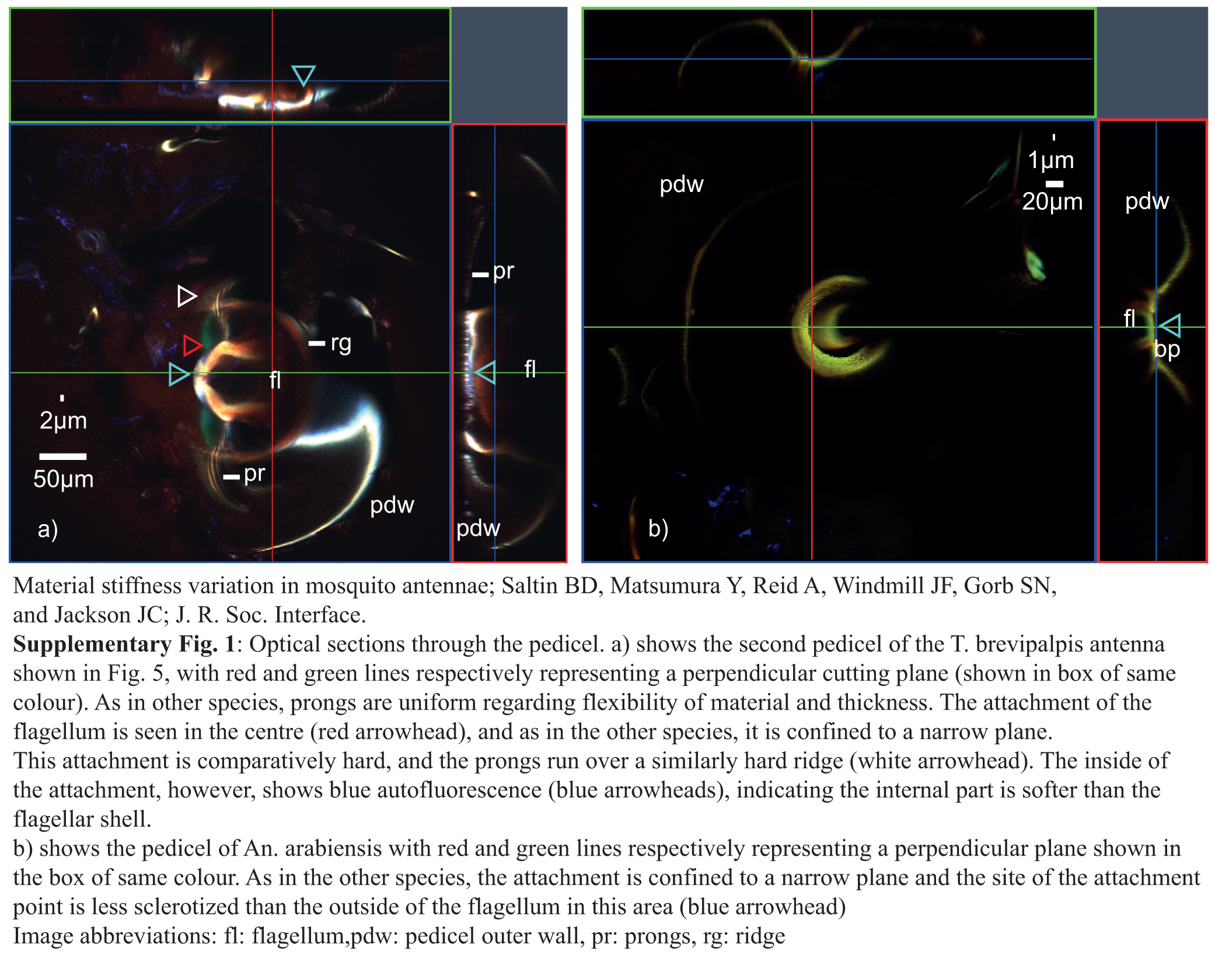

Supplement: Figure S1 [file rsif20190049supp1.tif]

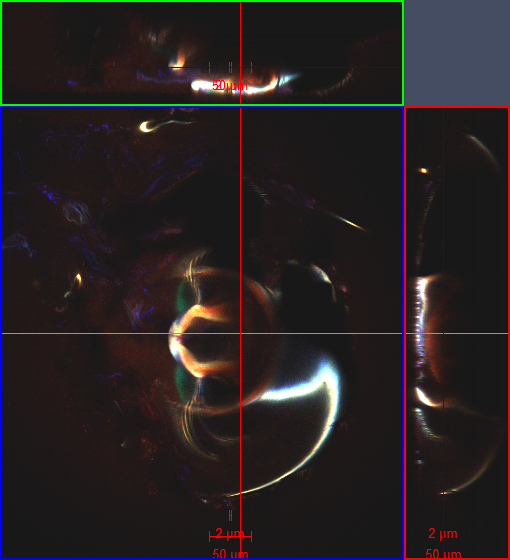

Supplement: Figure S1a [file rsif20190049supp2.tif]

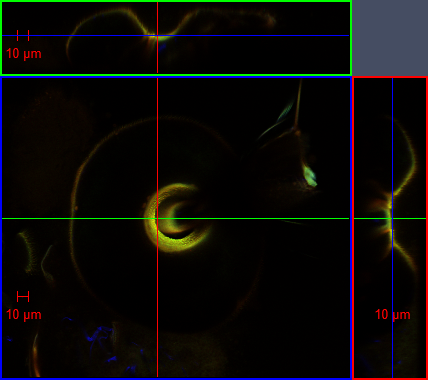

Supplement: Figure S1b [file rsif20190049supp3.tif]

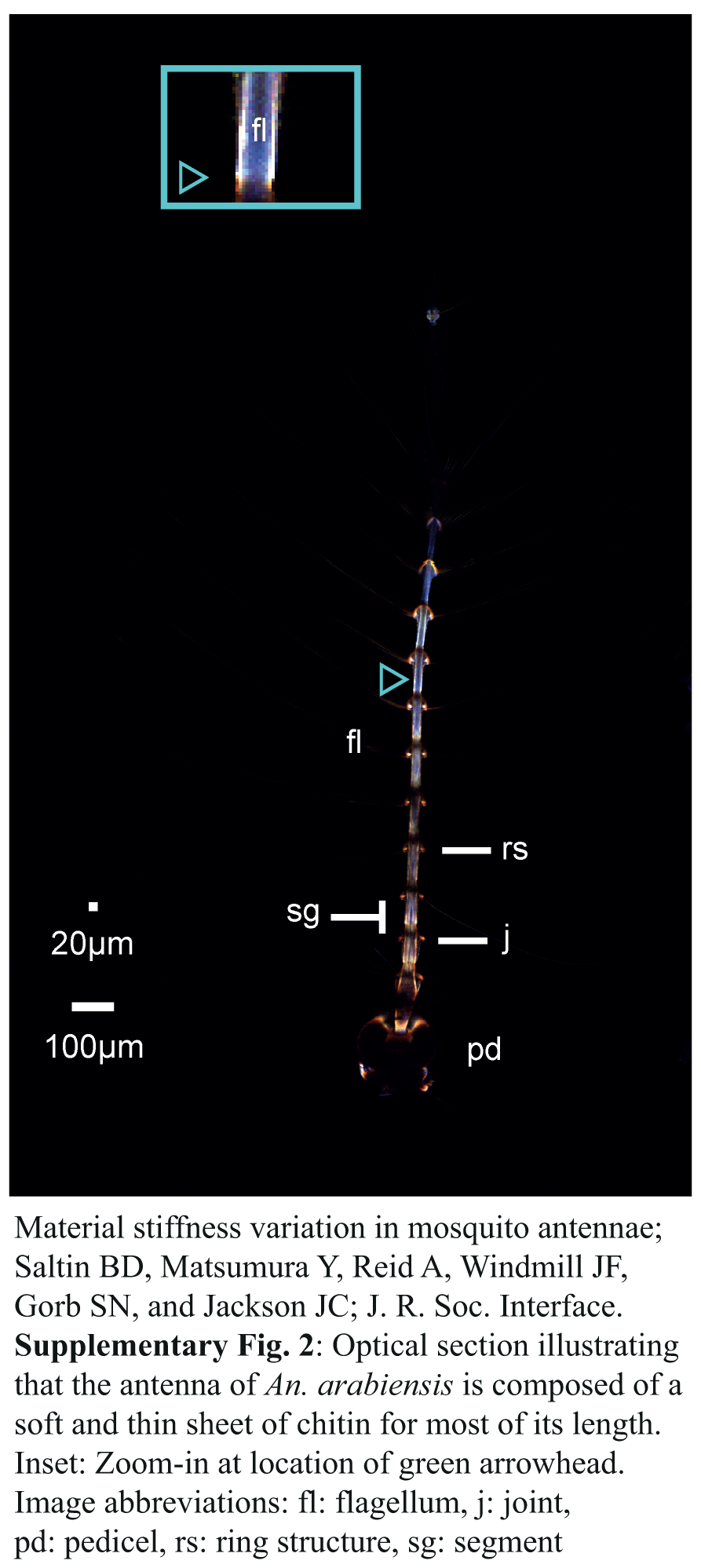

Supplement: Figure S2 [file rsif20190049supp4.tif]

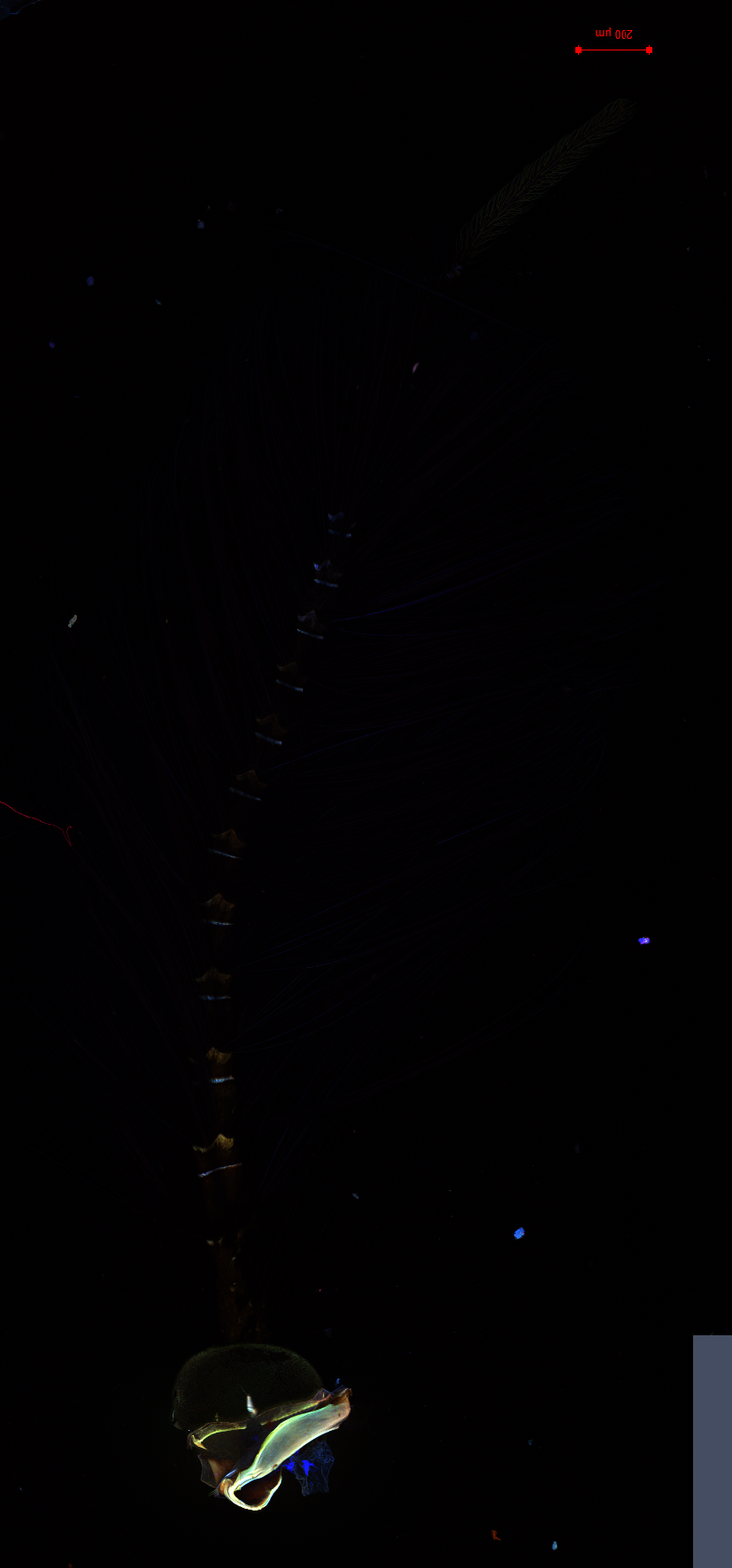

Supplement: Figure S3a [file rsif20190049supp5.tif]

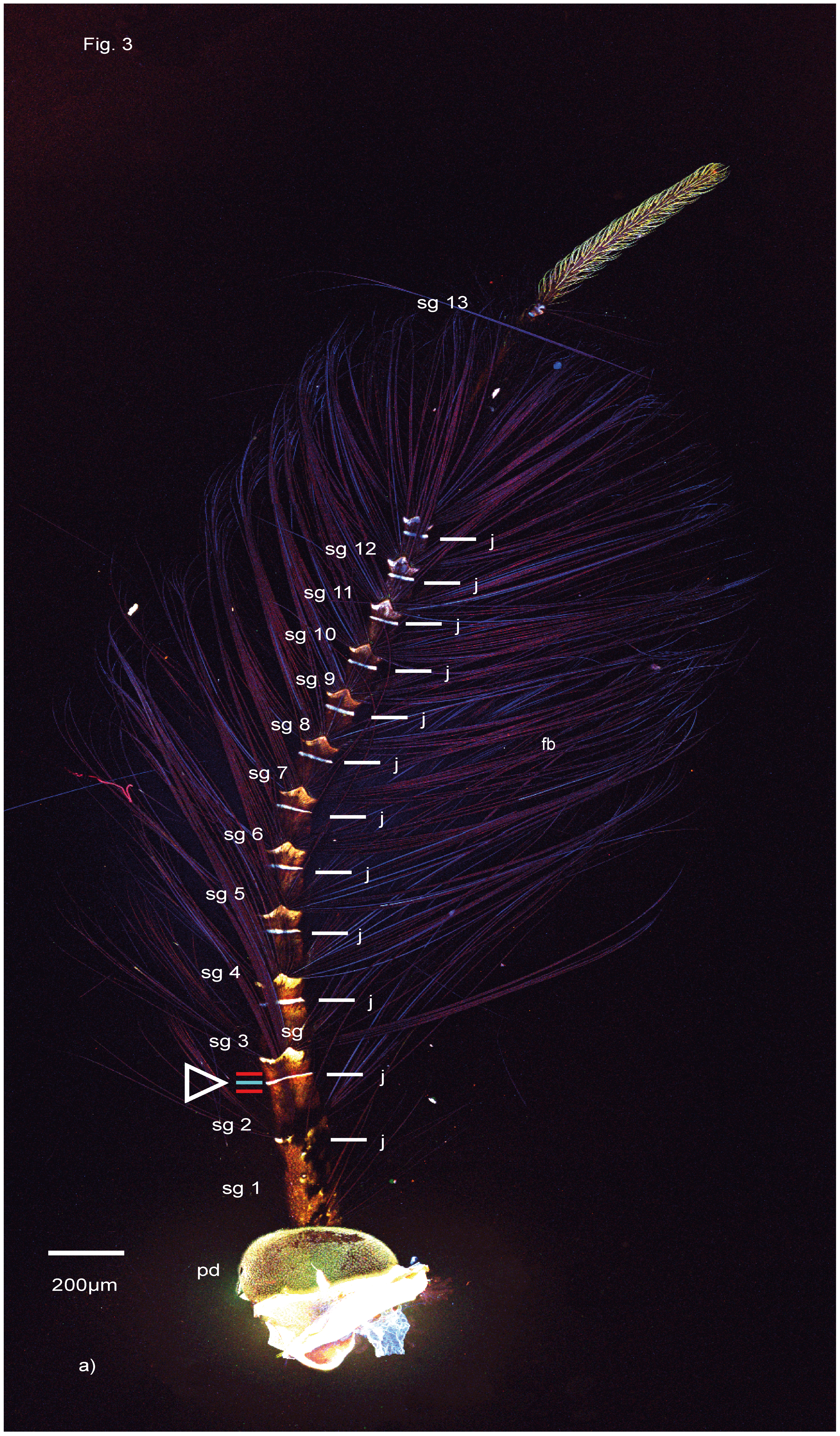

Supplement: Figure S3a - high resolution [file rsif20190049supp6.tif]

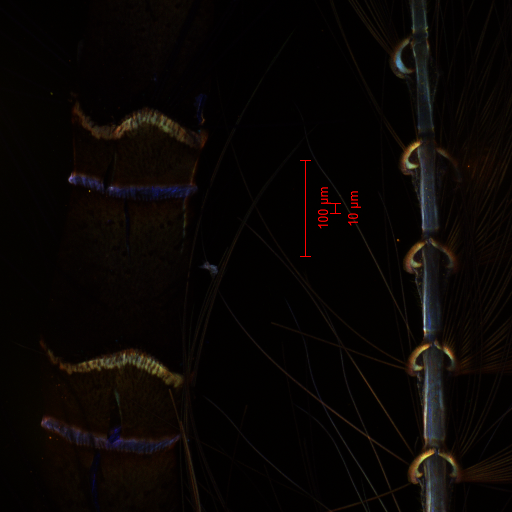

Supplement: Figure S3b&c [file rsif20190049supp7.tif]

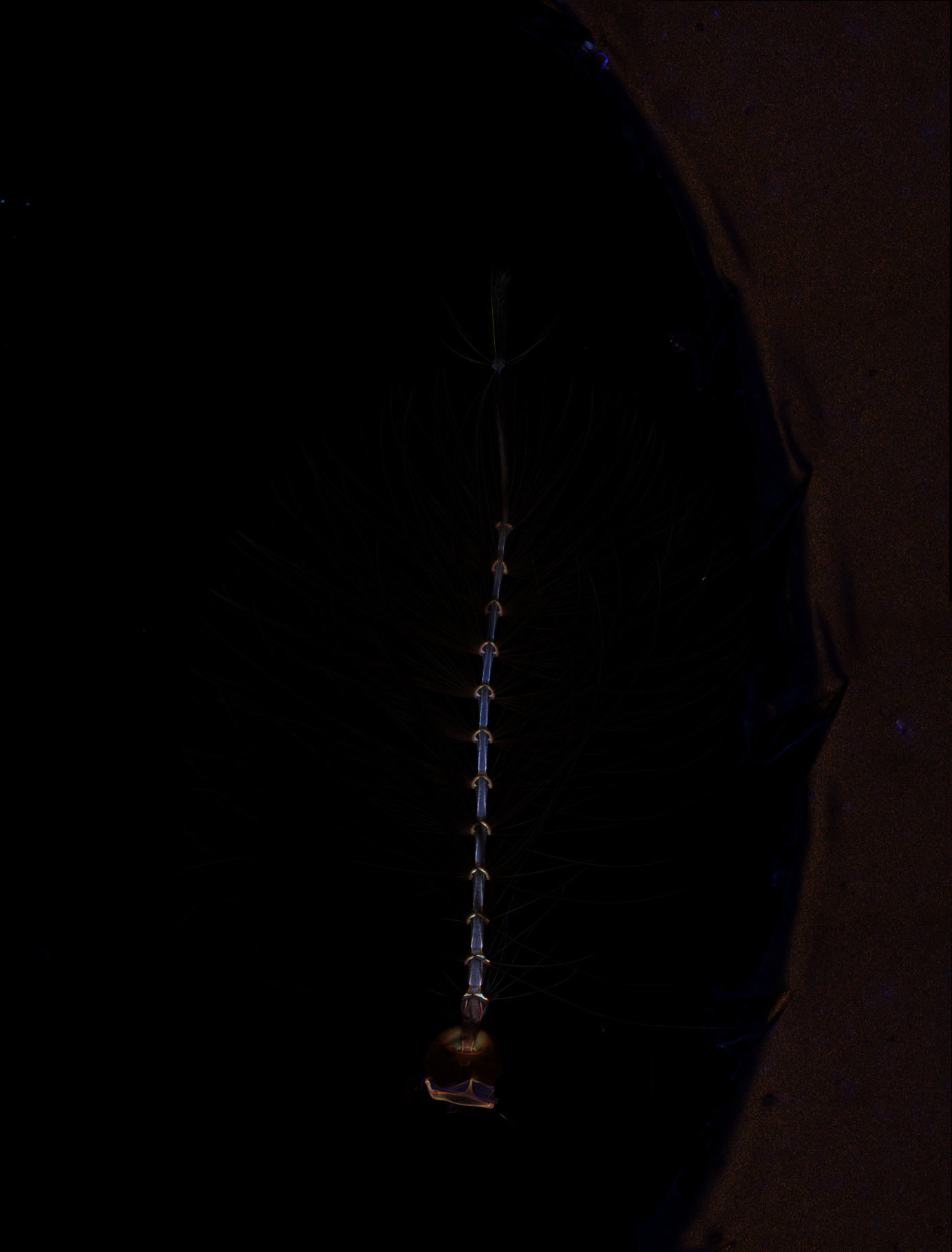

Supplement: Figure S3d [file rsif20190049supp8.tif]

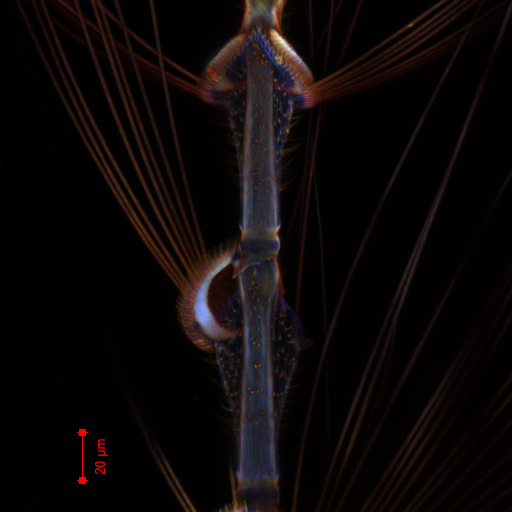

Supplement: Figure S4 [file rsif20190049supp9.tif]

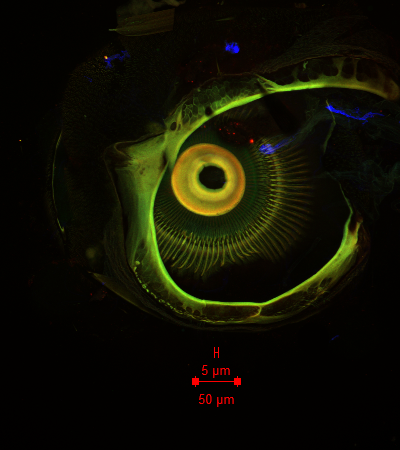

Supplement: Figure S5 [file rsif20190049supp10.tif]
