# Supplementary material for: Material stiffness variation in mosquito antennae
Source: J R Soc Interface. 2019 May 15;16(154):20190049. doi: 10.1098/rsif.2019.0049 (PMC6544878; doi:10.1098/rsif.2019.0049)
Supplement: Table S1 [file rsif20190049supp11.docx]

|  | Stack settings | | | Pixel size  [µm] | CH1  405nm  BP  420-480 | CH2  488nm  LP  490 | CH3  555nm  LP  560 | CH4  639nm  LP  640 |
| --- | --- | --- | --- | --- | --- | --- | --- | --- |
| Image title | Pixel-  Dwell  [µs] | Zoom | Nb | X/Y | %Power | %Power | %Power | %Power |
|  |  |  |  | Z | GAIN | GAIN | GAIN | GAIN |
| ^1^Fig. 3a:  *T. brevipalpis*  male antenna | 3.15 | 0.5 | 84* | 2.501 | 25% | 10% | 15% | 30% |
|  |  |  |  | 2.500 | 660 | 615 | 639 | 695 |
| ^1^Fig. 3b & c are of  *T. brevipalpis*  and *An. arabiensis*  for comparison | 3.15 | 1.2 | 34 | 1.042 | 20% | 3% | 20% | 22% |
|  |  |  |  | 2.983 | 637 | 665 | 600 | 698 |
| ^1^Fig. 3d & Supplemen-tary Fig 2:  *An. arabiensis*  male antenna | 3.15 | 0.7 | 68* | 1.786 | 15% | 2% | 15% | 15% |
|  |  |  |  | 2.500 | 657 | 656 | 568 | 695 |
| ^2^Fig. 4:  Detailed image of *An. Arabiensis* antenna | 3.15 | 1.5 | 81 | 0.417 | 20% | 3% | 20% | 30% |
|  |  |  |  | 0.630 | 658 | 649 | 604 | 708 |
| ^2^Fig. 5: Open pedicel of  *T. brevipalpis* | 3.26 | 0.6 | 120 | 1.186 | 15% | 3% | 15% | 20% |
|  |  |  |  | 0.700 | 491 | 632 | 593 | 660 |
| ^2^ Supplemen-tary Fig. 1a: Pedicel optical section of  *T. brevipalpis* | 3.26 | 0.6 | 172 | 1.186 | 15% | 3% | 15% | 20% |
|  |  |  |  | 0.700 | 599 | 621 | 592 | 657 |
| ^2^ Supplemen-tary Fig. 1b: Pedicel optical section of  *An. arabiensis* | 3.26 | 0.6 | 120 | 1.186 | 15% | 3% | 15% | 20% |
|  |  |  |  | 0.700 | 599 | 621 | 592 | 657 |

Material stiffness variation in mosquito antennae; Saltin BD, Matsumura Y, Reid A, Windmill JF, Gorb SN, and Jackson JC; J. R. Soc. Interface.

**Supplementary Table** **1**: CLSM settings listed by image. CH: Channel, BP: Band Pass. LP: Low Pass. Nb: Number of images per stack. Asterisk indicates multiple image tiles with an XY overlap of 15% that are stitched together into one image. The digital gain was 1 and no digital offset was used.

**Objectives used: ^1^Planapochromat 10x/0.45 M27. ^2^Planapochromat 20x/0.8 M27.**

All pinhole sizes were set as one Airy.
